# Acute Necrotizing Encephalopathy in a Child With RANBP2 Mutation: A Case of Unanticipated Full Resolution

**DOI:** 10.7759/cureus.91122

**Published:** 2025-08-27

**Authors:** Fadi Busaleh, Moayad Alshaqaq, Albatul R Bosaleh, Abdullah Bin Abd, Feras M Alharbi, Hussain Qallaf, Alyas K Alsaleh

**Affiliations:** 1 Pediatric Neurology/General Pediatrics, Maternity and Children Hospital, Al-Ahsa, SAU; 2 Medicine, Vision College, Al-Ahsa, SAU; 3 General Surgery, Al-Omran Hospital, Al-Ahsa, SAU; 4 Emergency Medicine, King Fahad General Hospital, Jeddah, SAU; 5 Emergency Medicine, Qatif Central Hospital, Qatif, SAU

**Keywords:** acute encephalopathy, acute necrotizing encephalopathy, encephalopathy, immune modulation therapy, ranbp2

## Abstract

Acute necrotizing encephalopathy (ANE) is a rare, rapidly progressive pediatric encephalopathy, often triggered by viral infections such as influenza A (H1N1), and typically characterized by bilateral thalamic involvement with high morbidity and mortality. We present a case of a previously healthy two-year-old girl who developed fever and respiratory symptoms, followed by sudden neurological deterioration and shock. Initial investigations revealed markedly elevated liver enzymes with normal cerebrospinal fluid analysis. Nasopharyngeal swabs tested positive for H1N1. Brain magnetic resonance imaging (MRI) revealed symmetric lesions involving the thalami and brainstem. Genetic analysis identified a heterozygous pathogenic mutation in the RANBP2 gene. Based on the clinical presentation, virologic findings, and characteristic imaging features, a diagnosis of ANE was established. The patient was promptly treated with intravenous immunoglobulin and high-dose corticosteroids, resulting in full neurological recovery within six weeks. This case underscores the importance of the early recognition and diagnosis of ANE and highlights the potential for favorable outcomes with timely immunotherapy, even in the presence of a RANBP2 mutation.

## Introduction

Acute necrotizing encephalopathy (ANE) is a rare, fulminant encephalopathy predominantly affecting children. It is characterized by multifocal and symmetric brain lesions primarily involving the thalami, brainstem, cerebellum, and subcortical white matter. Initially described in East Asia in the 1990s, ANE has since been reported worldwide [1].

It is most commonly associated with viral infections, particularly influenza A (H1N1), although other pathogens such as herpesviruses and SARS-CoV-2 have also been implicated [2,3]. The underlying pathogenesis is believed to involve a cytokine storm, with elevated levels of interleukin-6 and tumor necrosis factor-alpha triggering blood-brain barrier disruption, vascular endothelial damage, and subsequent necrosis of brain tissue [4]. Clinically, ANE presents with an abrupt onset of fever, seizures, altered consciousness, and often rapid neurological deterioration. Neuroimaging typically reveals bilateral thalamic lesions, which are considered a radiological hallmark of the condition [5]. Despite established clinical and radiological criteria, the diagnosis of ANE remains challenging due to its rarity and overlapping symptoms with other encephalopathies. We report a case of ANE in a two-year-old girl, highlighting the challenges in its diagnosis and the patient's favorable outcome.

## Case presentation

A previously healthy two-year-old girl presented to the emergency department at Maternity and Children Hospital in Al-Ahsa, Saudi Arabia, with a three-day history of fever associated with symptoms of an acute viral respiratory illness. On the third day of illness, she experienced sudden neurological deterioration with a decreased level of consciousness and hypotensive shock. She was admitted to the pediatric intensive care unit (PICU) with a working diagnosis of sepsis to rule out meningitis.

Shortly after admission, she developed generalized tonic-clonic seizures that were controlled after loading doses of anticonvulsants. She was intubated and mechanically ventilated. Empirical treatment with vancomycin and ceftriaxone was started, along with levetiracetam as maintenance medication for seizure control. Initial laboratory investigations revealed significantly elevated liver transaminases, leukopenia, and thrombocytopenia. Nasopharyngeal swabs were positive for H1N1 and influenza A viruses. Blood and cerebrospinal fluid (CSF) cultures were negative, and CSF analysis was unremarkable (Table 1).

**Table 1:** Patient's laboratory findings. CBC: complete blood count; WBC: white blood cell; ESR: erythrocyte sedimentation rate; ALT: alanine aminotransferase; AST: aspartate aminotransferase; ALP: alkaline phosphatase; LDH: lactate dehydrogenase; PT: prothrombin time; PTT: partial thromboplastin time; INR: international normalized ratio; H1N1: influenza A virus subtype H1N1; RANBP2: RAN-binding protein 2

Category	Test	Patient result	Reference range
CBC	WBC	4.28 × 10³/µL	6.0–17.0 × 10³/µL
	Hemoglobin	12.7 g/dL	10.5–13.5 g/dL
	Platelet	102 × 10³/µL	150–400 × 10³/µL
	ESR	10 mm/hr	0–10 mm/hr
Biochemistry	Random serum glucose	93 mg/dL	74–127 mg/dL
	Creatinine	27 µmol/L (0.31 mg/dL)	17.7–35.4 µmol/L
	Urea	4.3 mmol/L	1.8–6.0 mmol/L
	Calcium	2.41 mmol/L	2.1–2.7 mmol/L
	Magnesium	0.78 mmol/L	0.62–1.02 mmol/L
	Sodium	138 mmol/L	136–145 mmol/L
	Potassium	4.22 mmol/L	3.5–5.0 mmol/L
	Chloride	106 mmol/L	98–106 mmol/L
	Phosphorus	1.81 mmol/L	1.45–2.10 mmol/L
	LDH	583	200–600 U/L
	ALT	180 U/L	5–45 U/L
	AST	201 U/L	10–60 U/L
	ALP	188 U/L	Up to 350 U/L
	Total bilirubin	0.20 mg/dL	0.1–1.0 mg/dL
	Albumin	Not provided	3.5–5.0 g/dL
Coagulation profile	PT	20 sec	11–13.5 sec
	PTT	31 sec	25–35 sec
	INR	1.1	0.8–1.2
Cerebrospinal fluid (CSF)	CSF glucose	61 mg/dL	60%–80% of serum glucose
	CSF protein	34 mg/dL	15–45 mg/dL
	CSF WBC	Nil	0–5/mm³
	CSF RBC	Nil	0/mm³
	CSF culture	Negative	No growth
	CSF Gram stain	Negative	No organisms seen
Virology	H1N1	Positive	Negative
	Influenza A virus	Positive	Negative
Genetic testing	Whole exome sequencing	Positive for RANBP2	Negative

**Figure 1:**
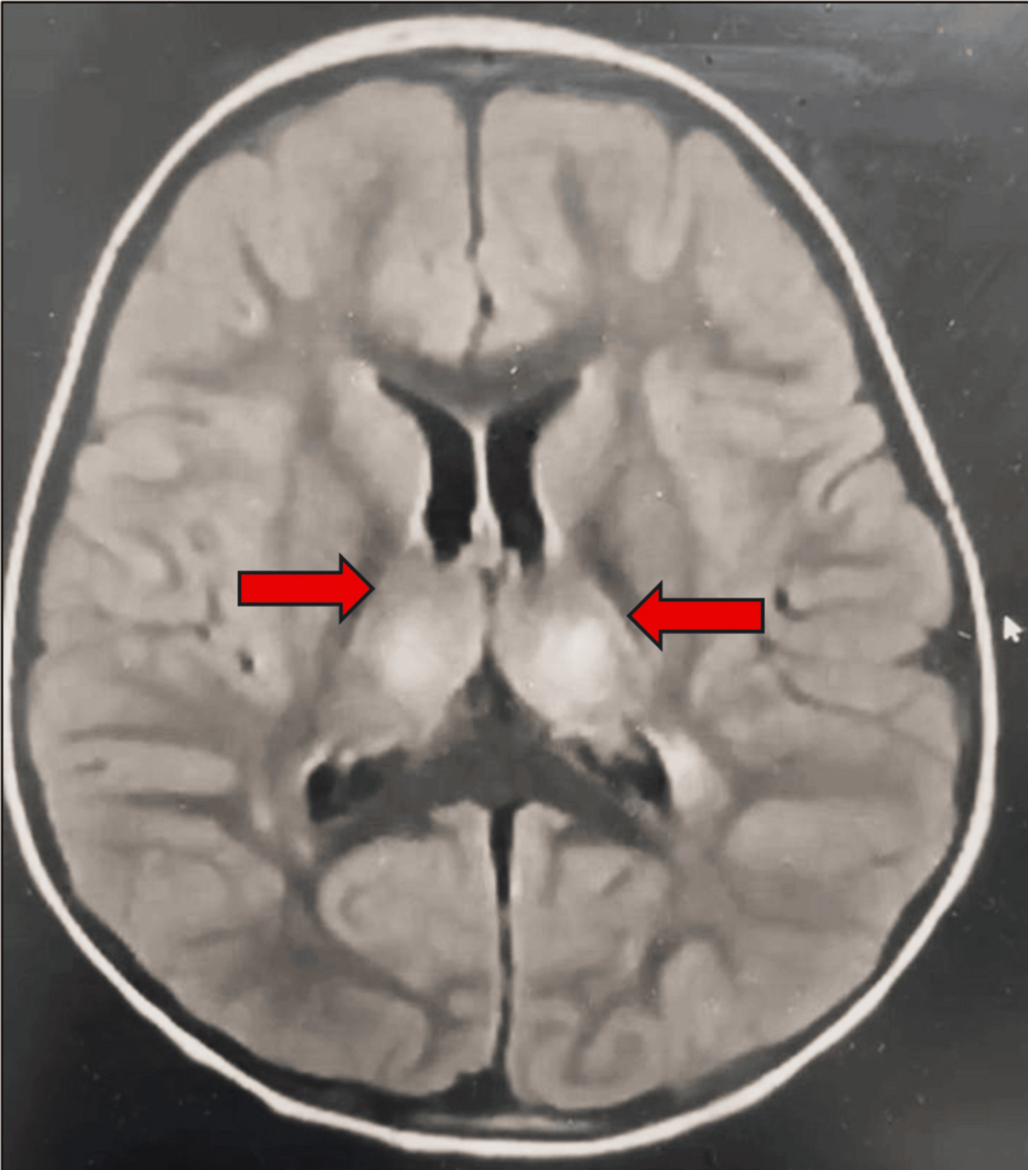
Axial brain MRI (FLAIR sequence) demonstrates bilateral symmetrical thalamic hyperintensities (red arrows). MRI: magnetic resonance imaging; FLAIR: fluid-attenuated inversion recovery

By the second day in PICU, upon tapering sedation, neurological examination revealed absent gag reflex, aphonia, ophthalmoplegia, and severe generalized weakness, raising concern for brainstem involvement or a structural brain injury. Urgent brain magnetic resonance imaging (MRI) of the brain demonstrated bilateral symmetrical hyperintensities involving the thalami, hippocampi, and brainstem, with relative sparing of the cerebral white matter (Figure 1). These clinical and ancillary findings were consistent with ANE. After the diagnosis of ANE was made, she was promptly started on intravenous (IV) immunoglobulin (IVIG) (1 g/kg/day for two days), followed by high-dose methylprednisolone (30 mg/kg/day for five days).

During the first week of illness, the patient remained dependent on nasogastric feeding and mechanical ventilation due to severe neurological impairment. She exhibited flaccid quadriparesis, with limb power graded at 1/5 in all extremities, and showed multiple cranial nerve deficits, including aphasia, aphonia, ophthalmoplegia, and dysphagia.

By the end of the first week following the initiation of immunotherapy, she demonstrated mild neurological improvement. She was successfully extubated, and her bulbar signs-particularly ophthalmoplegia and aphonia-began to improve. However, dysphagia and dysphonia remained significant, necessitating continued nasogastric feeding. Her limb strength also showed gradual recovery, improving to approximately 2-3/5.

During the third week, her neurological status continued to improve. She began to tolerate small amounts of soft food and sips of liquids orally. Her speech was slurred but more intelligible. Motor strength showed progressive improvement. By the end of the fourth week, she had returned to full oral intake and was speaking with good articulation. Limb power had improved to 4/5.

**Figure 2:**
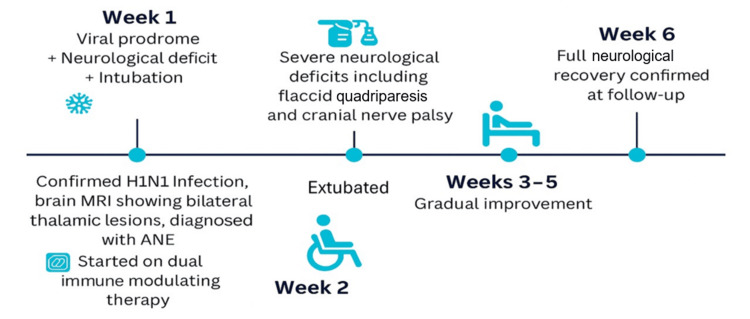
The visual timeline summarizes the progression of clinical events and highlights the patient's final neurological outcome.

By the fifth week from initial presentation, she had regained full motor function and normal cranial nerve function. She was able to walk independently, feed herself, and communicate normally. At the sixth week, she was seen in the pediatric neurology clinic for follow-up and was found to have returned to her pre-illness neurological baseline. The timeline of the clinical course is illustrated in Figure 2.

## Discussion

ANE is a rare, severe, and rapidly progressive neurological disorder, predominantly affecting otherwise healthy children following a febrile viral illness [1]. It is categorized as a para-infectious encephalopathy, meaning it arises as an immune-mediated complication following a systemic viral infection, most commonly influenza A or B, but also other viruses like human herpesvirus 6 [2,3]. The pathogenesis is thought to involve a dysregulated host immune response, leading to a "cytokine storm" that causes blood-brain barrier disruption, systemic inflammation, and hepatic enzyme elevation without direct viral invasion of the central nervous system or significant CSF inflammation [2,6]. Recent models and clinical studies further suggest that interleukins, specifically IL-6 and TNF-α, play central roles in ANE progression and severity, making them potential targets for future biologic therapies [7]. Our patient's clinical presentation, characterized by a rapid onset of neurological symptoms, elevated transaminases, and distinctive neuroimaging findings, was highly consistent with the classic triad observed in ANE cases worldwide, often precipitated by specific infections such as H1N1 [3,5,8].

In the differential diagnosis, neuroimmunological conditions like acute disseminated encephalomyelitis (ADEM) or infectious conditions such as meningitis or encephalitis were considered. However, the absence of demyelinating features on MRI, the lack of CSF pleocytosis, and the rapid development of symmetrical lesions helped differentiate ANE from ADEM [9-11]. Furthermore, elevated liver enzymes and normal CSF parameters, particularly negative cultures, were crucial in ruling out infectious causes [8-10].

Management of ANE primarily focuses on modulating the inflammatory cascade. Immune-modulating therapy, typically with high-dose corticosteroids and IVIG, is the cornerstone [1]. Our patient received early administration of methylprednisolone and IVIG within the first 72 hours, mirroring the protocol used in a multicenter study from Saudi Arabia by Bashiri et al., which demonstrated a very high survival rate among patients receiving early immunotherapy [12]. Additionally, to cover treatable conditions such as biotin-thiamine-responsive basal ganglia disease (BTRBGD), thiamine (100 mg IV daily) and biotin (5 mg/kg/day) were administered. This approach aligns with local Saudi Arabian protocols that emphasize vitamin supplementation in pediatric encephalopathy of uncertain etiology [13-15].

Genetic testing ultimately revealed a pathogenic heterozygous RANBP2 mutation, confirming a known genetic susceptibility for recurrent or familial ANE [2,16]. This finding holds particular relevance in Middle Eastern populations, where consanguinity is common, underscoring the importance of routine genetic evaluation in pediatric encephalopathies [17].

The prognosis of ANE is highly variable, ranging from complete recovery to severe neurological sequelae or even death. International studies report significant mortality rates, sometimes as high as 30%. The worst possible outcomes for ANE patients include severe, irreversible neurological damage leading to persistent vegetative states, profound developmental delays, intractable seizures, or even death [18]. Factors influencing these outcomes are multifaceted and can include the severity of initial brain involvement, particularly the extent of lesions in critical brain areas like the brainstem, the rapidity of disease progression, and the presence of genetic predispositions such as RANBP2 mutations, which can lead to recurrent episodes [1,2,5,16].

Timely diagnosis and prompt, aggressive treatment are consistently identified as crucial prognostic factors for a more favorable outcome [11,18]. This strategy proved highly effective for our patient, who achieved full neurological recovery. This positive outcome is directly attributable to the rapid recognition of ANE and the prompt initiation of immune-modulating therapy, a key management strategy consistently validated by both international and local studies [11,12,18]. Even with the identification of a pathogenic RANBP2 mutation, which underscores a genetic susceptibility to ANE, our patient's complete recovery powerfully demonstrates that aggressive and early treatment can lead to optimal outcomes, even in genetically predisposed individuals [2,16,17].

Although ANE survivors commonly experience some impairments, as noted in a Saudi Arabian registry [11,19], our patient's lack of residual deficits highlights the potential for exceptional recovery. This remarkable outcome stems from comprehensive and prompt care, including the early implementation of neurorehabilitation. This strategy was crucial for our patient, aligning with evidence that early rehabilitative interventions enhance functional recovery and reduce long-term neurological deficits in pediatric ANE cases [11,20].

## Conclusions

Pediatric encephalopathy presents a complex diagnostic challenge due to its wide range of potential causes, including infectious, autoimmune, metabolic, and genetic disorders. In this case, the patient’s clinical deterioration following a viral illness, along with characteristic neuroimaging findings of bilateral thalamic lesions, led to the diagnosis of ANE. This was further supported by the identification of influenza A (H1N1) infection and a heterozygous RANBP2 mutation. Differentiating ANE from conditions such as viral encephalitis and ADEM was critical for appropriate management. Early recognition allowed for timely intervention with immune-modulating therapy-IVIG and high-dose corticosteroids-alongside supportive care, metabolic evaluation, and genetic testing. Despite the initial severity, the patient experienced full neurological recovery within six weeks. This case highlights the importance of early diagnosis and fast, coordinated care to improve outcomes in this rare and serious condition.
